# Airway Microbiota in Severe Asthma and Relationship to Asthma Severity and Phenotypes

**DOI:** 10.1371/journal.pone.0152724

**Published:** 2016-04-14

**Authors:** Qingling Zhang, Michael Cox, Zhike Liang, Folke Brinkmann, Paul A Cardenas, Rachael Duff, Pankaj Bhavsar, William Cookson, Miriam Moffatt, Kian Fan Chung

**Affiliations:** 1 National Heart & Lung Institute, Imperial College, London, United Kingdom; 2 Respiratory Biomedical Research Unit, Royal Brompton & Harefield NHS Trust and Imperial College London, United Kingdom; 3 State Key Laboratory of Respiratory Diseases, The First Affiliated Hospital, Guangzhou Medical University, Guangzhou, China; University of Dundee, UNITED KINGDOM

## Abstract

**Background:**

The lower airways harbor a community of bacterial species which is altered in asthma.

**Objectives:**

We examined whether the lower airway microbiota were related to measures of asthma severity.

**Methods:**

We prospectively recruited 26 severe asthma, 18 non-severe asthma and 12 healthy subjects. DNA was extracted from induced sputum and PCR amplification of the V3-V5 region of bacterial 16S rRNA gene was performed.

**Results:**

We obtained 138,218 high quality sequences which were rarefied at 133 sequences/sample. Twenty OTUs had sequences ≥1% of total. There were marked differences in the distribution of Phyla between groups (*P* = 2.8x10^-118^). Bacteroidetes and Fusobacteria were reduced in non-severe and severe asthmatic groups. Proteobacteria were more common in non-severe asthmatics compared to controls (OR = 2.26; 95% CI = 1.94–2.64) and Firmicutes were increased in severe asthmatics compared to controls (OR = 2.15; 95%CI = 1.89–2.45). Streptococcal OTUs amongst the Firmicutes were associated with recent onset asthma, rhinosinusitis and sputum eosinophilia.

**Conclusions:**

Sputum microbiota in severe asthma differs from healthy controls and non-severe asthmatics, and is characterized by the presence of *Streptococcus* spp with eosinophilia. Whether these organisms are causative for the pathophysiology of asthma remains to be determined.

## Introduction

Asthma is a common airway condition characterized by airway inflammation and intermittent episodes of airflow obstruction. That asthma is a syndrome consisting of different phenotypes has been recognised for a long time by clinicians [1]. Features such as the onset of asthma either in childhood or during later years, the presence of atopy, the effect of upper respiratory tract viral infections in inducing exacerbations of asthma, asthma induced by ingestion of aspirin or non-steroidal anti-inflammatory agents, and the concomitant presence of comorbidities such as rhinosinusitis or obesity, have been recognised as important clinical features that delineate certain asthma clusters [1, 2]. In addition, asthma may be categorised by the severity of chronic symptoms and by the occurrence of recurrent exacerbations of asthma despite treatment with maximal asthma medications including corticosteroids [3]. T-helper type 2 cells [Th2] are recognised as an important underlying mechanism of a specific asthma phenotype that is associated with high levels of circulating IgE and atopy, and eosinophilia [4, 5] The presence of eosinophils in sputum samples has been known to be associated with asthma and has been used to indicate a good therapeutic response to inhaled or oral corticosteroid therapy [6, 7]. On the other hand, those with neutrophilic inflammation have been linked to corticosteroid insensitivity and in some instances to bacterial infections and colonisation [8, 9].

Consistent epidemiological indications of the importance of the microbiome in asthma include the protection afforded by a rich microbial environment in early life [10, 11], observations that the bronchial tree contains a characteristic flora that is disturbed by the presence of pathogens in asthma [12–14], birth cohort studies showing that the presence of the same pathogens in throat swabs predicts the later development of asthma [15], recognition that these bacteria have consistently been associated with exacerbations of asthma [16], and evidence for the efficacy of antibiotics in the treatment of asthma and related syndromes [17–19]. In 27% of asthmatic patients presenting with an exacerbation of asthma, bacteria have been cultured from sputum samples, with *Streptococcus pneumoniae*, *Streptococcus pyogenes*, *Staphylococcus aureus*, *Moraxella catarrhalis* and *Haemophilus influenzae* being the most frequent bacteria isolated [20]. This spectrum of bacterial species was also isolated from induced sputum samples in 15% of patients during a stable period of asthma [8]. In addition, other species such as *Mycoplasma pneumoniae* and *Chlamydophila pneumoniae* have also been detected in patients with asthma [21]. Using a terminal restriction fragment length polymorphism profiling for bacterial community analysis, another study showed that the dominant species within the airway bacterial community in sputum samples from treatment-resistant asthma was *Moraxella catarrhalis* or a member of the *Haemophilus* or *Streptococcus* genera [22].

16S ribosomal RNA microarray rather than conventional culture techniques have been used recently to detect operational taxonomic units in lower airway epithelial samples. This technique has led to the detection of a microbiome in the lower airways of normal subjects that has hitherto been considered to be a sterile environment. In addition, airways of asthmatic subjects were reported to show a different microbiota compared to non-asthmatic subjects in terms of an increase in Proteobacteria particularly in *Haemophilus*, *Moraxella*, and *Neisseria* spp, with a reduction in Bacteroidetes [12]. Another study confirmed the increase in bacterial load particularly Proteobacteria in the lower airways of asthmatics, and the total bacterial burden and diversity correlated with bronchial hyperresponsiveness [13]. In induced sputum, a higher proportion of Proteobacteria with lesser content of Firmicutes and Actinobacteria in asthmatics compared to non-asthmatics was reported. By contrast, no differences in airway bacterial load were observed between subjects with asthma and normal control subjects as assessed by 16S rRNA copy number [23]. Neonates colonized in the hypopharyngeal region with *S*. *pneumoniae*, *H*. *influenzae* or *M*. *catarrhalis*, or with a combination of these organisms were at increased risk for recurrent wheeze and asthma early in life [15]. In neonates from the tropics, the increased prevalence of *Haemophilus* and *Staphylococcus spp* was proposed to contribute to wheezing illnesses [14]. Whether they contribute to asthma pathogenesis or whether they are altered as a consequence of asthma is at present unresolved [24]. However, the role of pathogens in causing disease and the actions of commensal bacteria in maintaining a healthy mucosa in the gut are well-recognised [25].

Previous studies of the airway microbiome showed that that the airways of moderate asthmatics contained an excess of Proteobacteria [12, 26] compared to normal controls, but asthma is heterogeneous in its clinical presentation and severity [3, 27] and it is possible that the airways of severe asthmatics have a different microbiota. In stable asthma patients with more severe disease, up to half had one or more bacteria cultured from sputum, with *H*. *influenzae*, *P*. *aeruginosa* and *S*. *aureus* being the most common [28]. It is therefore extremely valid and important to examine the respiratory microbiota in patients with severe asthma and determine whether the airway microbiota is altered compared to mild-moderate asthma as these differences could be important in determining both the pathophysiology and treatment of severe asthma. Therefore, we have determined whether patients with severe asthma have an altered microbiota compared to non-severe asthma measured by high throughput sequencing of 16S ribosomal RNA gene. More importantly, we also determined whether the altered microbiota were associated with any particular phenotype of asthma.

## Methods

This study was approved by the Ethics Committee of the Royal Brompton & Harefield NHS Trust. All patients provided written informed consent to participate in this study by signing the consent form that was approved by the Ethics Committee of the Royal Brompton & Harefield NHS Trust.

### Subjects

All non-asthmatic subjects were healthy volunteers without any disease with normal spirometric results (Table 1). Patients with severe asthma were recruited from the Severe Asthma clinic at the Royal Brompton Hospital, London, over a 6-month period. Patients with severe asthma needed either continuous or near-continuous oral corticosteroids, high-dose inhaled corticosteroids, or both to achieve a level of mild-to-moderate persistent asthma, and by 2 or more minor criteria [29]. All patients had been attending the clinic for at least 6 months and had undergone a Severe Asthma Protocol for confirmation of the diagnosis of severe asthma [30]. Patients with non-severe asthma had controlled asthma as defined by no daytime or nocturnal symptoms, no limitation of activities, minimal use of rescue medication while using up to 2,000 μg/d or equivalent of inhaled beclomethasone. Current smokers and ex-smokers of greater than 5 pack years were excluded. Patients with an exacerbation of asthma within the last 4 weeks or with a respiratory tract infection requiring antibiotic treatment within 6 weeks were excluded.

**Table 1:** Characteristics of the subjects^1^.

Parameters	Healthy	Non-severe asthma	Severe asthma
Number	12	18	26
Age (years)	35.4±10.3	45.2±16.0	47.9±10.9**
Gender (male:female)	1:11	10:8	9:17
BMI (kg/m^2^)	24.5±5.7	26.9±4.7	28.9±6.2*
Duration of asthma (years)	0	29.7±19.3	22.6±16.5
Ex-Smoker (n)	0	3	0
Rhinosinusitis (n)	0	4	8
Atopy ^4^ (n)	0	9	24
IgE IU/L^2^	48.4 (26–80)	112.2 (14–510)**	148.4 (2–4335)** ¶
Daily prednisolone (mg.day^-1^)	0	0	17.5±9.3
Inhaled corticosteroids^3^ (μg.day^-1^)	0	1453±563	2348±1140¶¶
FEV_1_ (L)	3.78±0.63	2.88±0.94	2.25±0.60
FEV_1_ (% predicted)	98.7±11.0	87.0±15.3	76.7±15.9
FVC (L)	4.69±0.59	3.89±0.98	3.25±0.74
FVC (% predicted)	101.8±6.3	99.8±14.7	92.0±11.7
Bronchodilator reversibility^5^ (%)	Not done	14.4±10.0	17.9±12.1
Blood eosinophils (per μl)	0.10±0.0	0.28±0.17**	0.40±0.51
Sputum eosinophils (%)	0.4±0.4	6.9±12.4	19.4±26.9** ¶
Sputum neutrophils (%)	49.6±27.6	45.7±24.8	45.0±27.4

^1^ Data shown as mean ± SD unless otherwise denoted.

^2^ Data shown as geometric mean and range.

^3^ Inhaled corticosteroid expressed as beclomethasone propionate equivalent dose

^4^ Atopy defined as by the presence of positive skin-prick tests to at least one common aeroallergen.

^5^ Bronchodilator reversibility calculated as postbronchodilator FEV_1_-prebronchodilator FEV_1_/prebronchodilator FEV_1_ x 100.

BMI: Body mass index; FEV_1_: forced expiratory volume in one second; FVC: forced vital capacity.

*P<0.05 compared to healthy controls;

**P<0.01 compared to healthy controls;

^¶^*P*<0.05 compared to non-severe asthma;

^¶¶^*P*<0.01 compared to non-severe asthma.

### Lung function and atopic status

FEV_1_ and forced vital capacity (FVC) were measured using a spirometer (Erich Jaeger UK Ltd, Market Harborough, UK). Atopy was defined by the presence of positive skin-prick tests to at least one common aeroallergen including house dust mite, grass and tree pollen, cat dander, dog dander and Aspergillus.

### Sputum induction, cell count and microbiology

Sputum was induced by inhalation of an aerosol of sterile 3% saline solution and subsequently increasing to 4% and 5% during 3 periods of 2 minutes each after the subject has had pre-treatment with inhaled salbutamol [31]. Particular care was taken to avoid contamination with saliva and post-nasal drip by instructing subjects to rinse orally with water and to blow their nose after each inhalation. Sputum samples were collected into sterile pots. Peak flow measurements were made after each inhalation. The procedure was not carried out if the subject brought up spontaneously sputum and if the baseline post-bronchodilator FEV_1_ was less than 60% predicted or less than 1.0 litre. If there was a fall in peak flow of 20% or more or if symptoms occurred during the procedure, the induction was stopped.

For sputum bacteriological culture, an aliquot of induced sputum was selected using a positive displacement pipette and used for quantitative bacteriological culture [30]. Sputum plugs were selected and one portion was used to perform differential cell counts. To perform differential cell counts, di-thiothreitol was added to the sputum plug and mixed vigorously on a plate shaker to solubilize the sputum. Cytospins were then prepared, and differential cell counts obtained.

For sputum 16S RNA assay, sputum plugs weighing more than 0.1g were selected and placed in a 1.5 ml sterile polypropylene centrifuge tube for DNA extraction.

### DNA extraction and PCR amplification and sequencing

DNA extraction was performed using the QIAmp DNA Mini Kit extraction kit, modified to include a bead-beating step. The method proceeded as per manufacturer’s instruction until the end of the Proteinase K incubation, when the sample and lysis buffer were transferred to lysing matrix E bead-beating tubes (LME tubes; MP Biomedicals), and beaten in a Precellys bead-beater (Bertin Technologies) for 2 cycles of 30 seconds at 6800 rpm. DNA was eluted from the QIAmp columns in 40 μl of nuclease free water and stored at -80°C until further use.

PCR amplification of the V3-V5 region of the 16S rRNA gene was performed using 1 μl of extracted DNA template. Reactions were performed in quadruplicate using primer pair 357F/926R (357F - CCTACGGGAGGCAGCAG, 926R - CCGTCAATTCMTTTRAGT), coupled with Roche 454 sequencing adaptors A (reverse primer) and B (forward primer) and a unique 12 base-pair barcode [32]. Replicates were combined and purified twice using Ampure XP (Beckman Coulter) bead purification, before quantification using the Quant iT kit (Life Technologies) and equimolar pooling. Thirty to fifty samples were pooled in each sequencing run on a Roche 454 Junior pyrosequencer, using the Lib-L kit and protocol (Roche Diagnostics Ltd).

### Data pre-processing

Raw sequence files from each run were combined prior to denoising and chimera removal in QIIME [33] using Ampliconnoise and Perseus [34]. Operational taxonomic units (OTUs) at 97% identity were picked using UCLUST [35], the most abundant sequences in each OTU selected as representatives and classified using the RDP classifier [36] retrained with the Silva database version 111 [37]. Random resampling of the OTU table to 133 reads was performed in order to ensure equal sequence depth of each sample, whilst maintaining the ability to discriminate between samples, resulting in 3458 analysed.

### Data analysis

Statistical analyses of phenotypic relationships were all carried out using IBM SPSS version 21. Asthma status was coded as non-asthmatic = 0, non-severe asthma = 1, and severe asthma = 2. The complex relationships between phenotypic variables were explored with Principal Components Analysis (PCA) in the FACTOR routine in SPSS. Missing values were replaced with the study mean for the relevant parameter. PCs with an eigenvalue >1 were retained in the analysis, and a varimax rotation was applied. Bacterial counts were log-normalised after adding 1 to each count (to return a log value of 0 when the count was 0) before parametric analyses. Analyses of community diversity were carried out in the R statistical environment using the Phyloseq and Vegan packages.

## Results

### Patient characteristics

The study population consisted of 56 subjects (12 normal subjects, 18 non-severe asthmatics and 26 severe asthmatics) (Table 1). Severe asthmatics had lower %FEV_1_ predicted (P<0.05) and higher sputum eosinophil counts (P<0.05), and higher BMI and atopy compared to non-severe asthma and normal subjects. 15 of the 26 severe asthma patients were on daily oral prednisolone using a mean dose of 17.5 mg per day.

Out of the 56 samples, potentially pathogenic bacteria were cultured from the sputum of 5 stable severe asthmatics (3 x *Haemophilus influenzae*, 1 x *Moraxella catarrhalis* and 1 x *Staphylococcus aureus*) and 5 stable non-severe asthmatics (1x, 2x and 2x respectively). No potentially pathogenic bacteria were cultured from the 12 healthy controls.

### PCA analysis of clinical variables

PCA was carried out with the most important phenotypic variables (Table 2). In order to avoid over-fitting of the model, single variables were chosen to represent highly correlated related parameters. Four Principal Components (PC1-4) with eigenvalues >1 and accounting for >10% of the variance were identified (Table 2). Asthma status (from normal to severe asthma) was most strongly associated with the first two PCs, PC1 and PC2 (Table 2).

**Table 2:** Principal Components Analysis of phenotypic variables.

	Component (% of total variance)			
	1 (24%)	2 (16%)	3 (15%)	4 (12%)
Diagnosis	0.663	0.525	0.191	0.273
FEV_1_ (% predicted)	-0.117	-0.515	-0.227	-0.520
Asthma duration	0.526		-0.113	0.567
Atopic status	0.773	0.351	0.213	
Rhinosinusitis	0.138		0.619	0.271
Body mass index	0.525			
Age	0.139		0.149	0.802
Inhaled corticosteroids	0.860	0.101		0.318
Salbutamol use	0.379	0.671	-0.174	
Long-acting β-agonist	0.836	0.118		0.364
Oral corticosteroids	0.202	0.895		
Sputum neutrophils			-0.750	0.247
Sputum eosinophils	-0.188	0.532	0.602	0.215
Blood eosinophils		-0.105	0.773	0.127

Principal Components accounting for <10% variance and vector coefficients < 0.1 are not shown.

PC1 was typified by asthma of relatively long duration, atopy, a relatively high BMI, the use of inhaled steroids and long-acting β-agonist and a weak negative association with % sputum eosinophils. PC2 was associated with poor lung function, oral corticosteroid use and sputum eosinophilia. PC3 was typified by relatively recent onset, nasal disease, blood and sputum eosinophilia, and relatively fewer neutrophils in sputum. PC4 was characterised by the relatively increased age of the patients, poorer lung function and treatment with inhaled corticosteroids and long-acting β-agonist.

### Operational taxonomic units (OTUs)

We obtained a total of 138,218 high quality sequences after de-noising and chimera removal. Following exclusion of singletons, 163 OTUs remained. Samples were rarefied at 133 sequences per sample for statistical analyses, such that a total of 7448 reads were used for subsequent analyses. Rarefaction with higher numbers of sequences reduced the numbers of subjects but did not change the principal findings of the study. Many of the OTUs were relatively uncommon, and we found 31 OTUs with numbers of sequences that were ≥0.5% of the total, 20 OTUs with sequences ≥1% of the total and 10 OTUs which made up ≥2% of the total. Because statistical power to detect associations to uncommon OTUs is limited, we confined downstream analyses to the 20 OTUs with sequences ≥1% of the total. We further excluded an OTU identified as Burkholderia (1.3% of total reads) because it was confined to three subjects and our kit controls indicated it to be a common contaminant.

**Fig 1:**
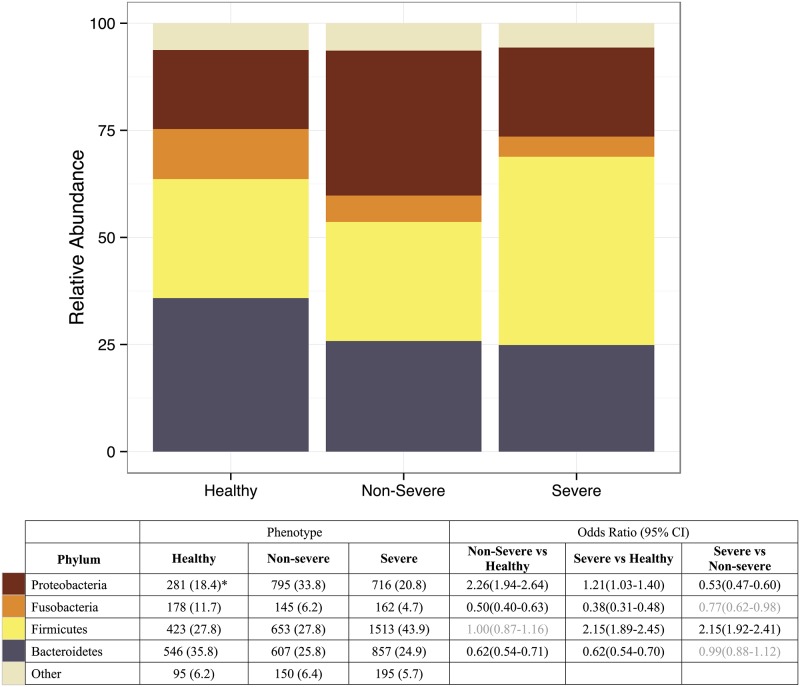
Distribution of bacterial Phyla in healthy controls and non-severe and severe asthmatics defined by sequencing of the 16S rRNA gene. The distribution of bacterial phyla from 12 healthy subjects, 18 non-severe asthmatics and 26 severe asthmatics are shown. Compared to healthy controls, non-severe asthmatics showed a reduction in the prevalence of Bacteroidetes and Fusobacteria and an increase in Proteobacteria (all *P*<10^−10^). Severe asthmatics showed an increase in Firmicutes compared to controls and non-severe asthmatics, together with reduction in Bacteroidetes and Fusobacteria compared to controls (all *P*<10^−10^). The distribution of bacterial phyla is given in the accompanying lower Table as Operational Taxonomic Unit (OTU) counts with % total in brackets. The legend gives the odds ratios for each comparison. The overall χ^2^ for differences in phyla between phenotypic groups = 557.7 (*P* = 2.8x10^-118^). *OUT counts (% total).

The most common phylum in the dataset was Firmicutes, containing 35.4% of sequences, followed by Bacteroidetes with 27.5% and Proteobacteria with 24.5%. There were marked differences in the distribution of Phyla between the three patient groups (χ^2^ = 530, 5 d.f.; *P* = 2.2x10^-112^) (Fig 1).

Comparison between healthy controls and patients with non-severe asthma showed a reduction in the prevalence of Bacteroidetes, containing OTUs identified predominately as Gram-negative anaerobes such as *Prevotella* spp. (Odds Ratio (OR) = 0.62; 95% confidence interval (CI) = 0.54–0.71) and Fusobacteria (OR = 0.50; 95%CI = 0.40–0.63) (Fig 1), whereas Proteobacteria, containing potential respiratory pathogens such as, *Neisseria* and *Moraxella* spp., were more common in the patients with non-severe asthma compared to controls (OR = 2.26; 95%CI = 1.94–2.64).

Bacteroidetes and Fusobacteria were also reduced in the severe asthma group compared to healthy controls (OR = 0.62; 95% CI = 0.54–0.70 and OR = 0.38; 95% CI = 0.31–0.48, respectively) (Fig 1). A minor increase in Proteobacteria was also seen in the severe asthmatics (OR = 1.21; 95% CI = 1.03–1.40) compared to non-asthmatic controls, but this was much less striking than the increase observed in Firmicutes, consisting predominately of streptococcal OTUs (OR = 2.15; 95% CI = 1.89–2.45). The increased presence of Firmicutes in severe asthma was also evident in comparison with the non-severe asthma group (OR = 2.15; 95% CI = 1.92–2.41) (Fig 1).

These results are consistent with earlier studies that show a reduction in Bacteroidetes and an increase in Proteobacteria in adults and children with asthma [12, 14]. Our present results also indicate that the microbiota of severe asthmatics differs from that of non-severe asthmatics, and is characterized by an increase in the prevalence of Firmicutes rather than Proteobacteria.

**Fig 2:**
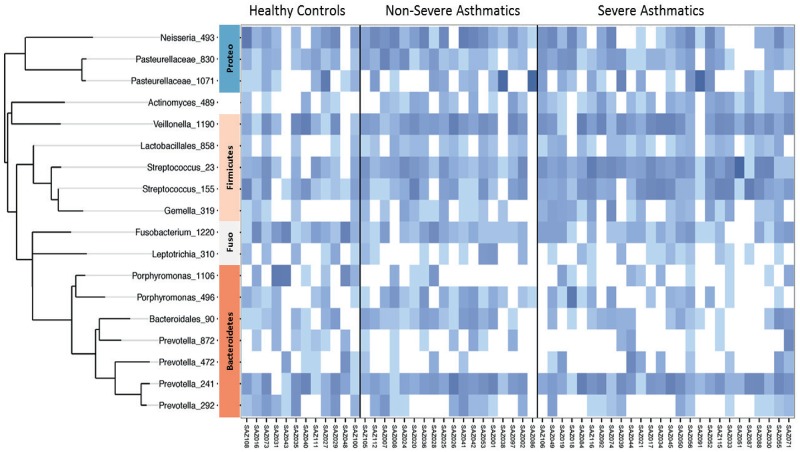
Heat map of operational taxonomic units (OTUs) found in the sputum of asthmatics and non-asthmatic subjects. The phylogenetic tree for the principal (>1% of total) OTUs is shown at the left. On the right, increasing depth of colour indicates relative abundance of the OTU in an individual sample. Major phyla are shown between the phylogenetic tree and the heat map. Proteo = Proteobacteria; Fuso = Fusobacteria.

We next looked for differences closer to the species level, defined by particular OTUs. These two Streptococcus OTUs were positively correlated with asthma severity (*Streptococcus*_23, r = 0.41, P = 0.002; and *Streptococcus*_15, r = 0.35, P = 0.009), and one Prevotella OTU showed a negative correlation (*Prevotella*_292, r = 0.36, P = 0.007). Phylogenetic analysis of these sequences was not able to further differentiate the Streptococcus OTUs into individual species. Fig 2 shows the heat map of OTUs found in the sputum samples from asthmatic and non-asthmatic subjects.

Repeating the PCA and including the individual counts for these three OTUs showed that PC3 (relatively recent onset asthma with eosinophilia and nasal disease) was most strongly related with the presence of the two Streptococcus OTUs (Table 3). Weaker relationships were seen between *Streptococcus*_23 and PC1 (atopic disease treated with inhaled corticosteroid and long-acting β-agonist), and *Streptococcus*_155 and PC2 (severe asthma with sputum eosinophilia). PC2 was also associated with lower counts of *Prevotella*_292.

**Table 3:** Principal Components Analysis of phenotypic variables including microbial associations.

	Component (% of total variance)			
	1 (21%)	2 (15%)	3 (13%)	4 (12%)
Diagnosis	0.661	0.533	0.259	0.239
FEV_1_ (% predicted)	-0.157	-0.471		-0.624
Asthma duration	0.668		-0.237	0.319
Atopic status	0.577	0.449	0.262	
Rhinosinusitis	0.111		0.311	0.595
Body mass index	0.225	0.289		0.102
Age	0.388			0.661
Inhaled corticosteroids	0.934	0.173		
Salbutamol use	0.362	0.693	-0.111	
Long-acting β-agonist	0.923	0.175		
Oral corticosteroids		0.897	0.207	
Sputum neutrophils	0.176		-0.659	-0.243
Sputum eosinophils	-0.134	0.326	0.471	0.525
Blood eosinophils			0.558	0.487
Streptococcus_155	0.106	0.334	0.707	
Streptococcus_23	0.342		0.671	
Prevotella_292	-0.120	-0.404	-0.111	-0.145

Principal Components accounting for <10% variance and vector coefficients < 0.1 are not shown.

**Fig 3:**
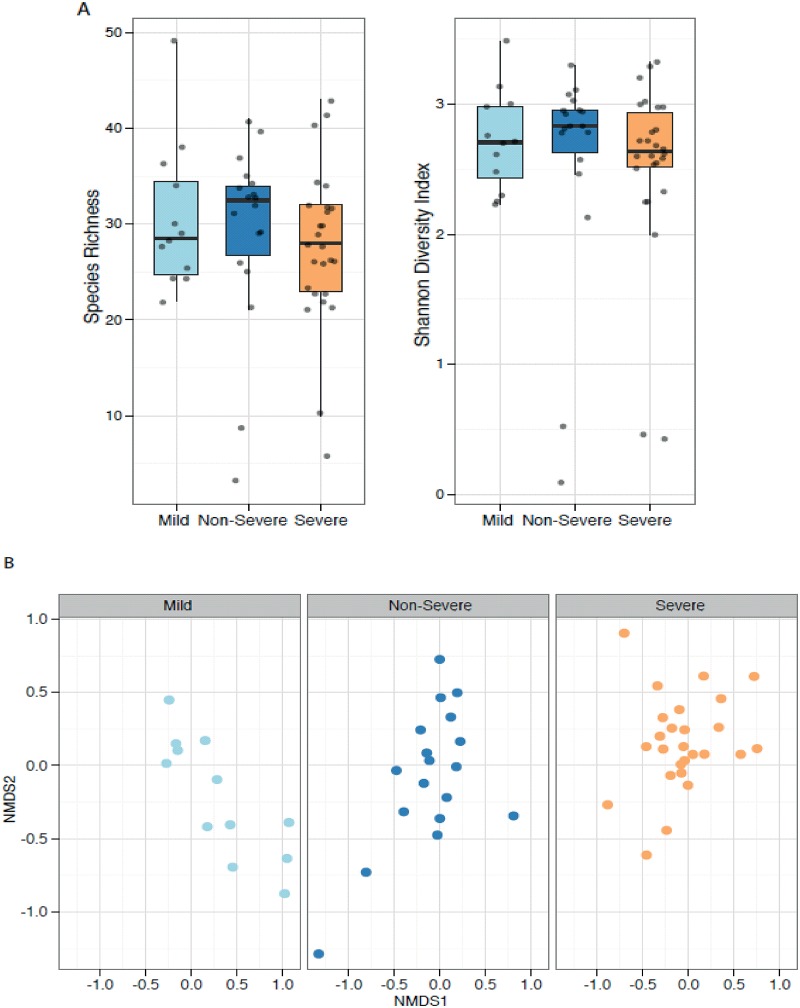
Diversity of microbial communities in healthy, non-severe and severe asthmatics. A. Boxplot of alpha diversity measures Species Richness and Shannon’s Diversity Index, which are not significantly different between groups. B Non metric multidimensional scaling of Bray Curtis distance split by group (stress 0.22). Here PERMANOVA (Adonis) indicates that community structure is significantly associated with asthma classification (P = 0.008) and this variable explains 6% of the variance.

Alpha diversity measures, species richness (the number of different OTUs), Pielou’s evenness (the skew or dominance of OTUs), Shannon and Inverse Simpson’s diversity indices, were calculated for each sample. There were no significant differences in alpha diversity between healthy, non-severe and severe asthmatics (Fig 3A). A Bray Curtis dissimilarity matrix was calculated and PERMANOVA (Adonis) used to demonstrate that community structure was significantly different between disease groups and they explained 6% of the variance in community structure (*P* = 0.008, Fig 3B).

## Discussion

This analysis of the microbiota in patients with non-severe and severe asthma is one of the most substantial reported so far. We defined severe asthma as patients who remained uncontrolled despite taking sufficient amounts of asthma medication including corticosteroids, often as systemic doses as well as via the inhaled route. Even in this modest number of subjects, we found clusters of severe asthma such as those with poor lung function, oral corticosteroid use and sputum eosinophilia. We found that there were marked differences in the distribution of Phyla between groups. Our findings in patients with non-severe asthma are strongly consistent with earlier studies showing an increase in Proteobacteria and a reduction in non-pathogenic commensals when compared to controls [12, 14, 26]. We discovered equally distinctive changes in the severe asthma group. Bacteroidetes and Fusobacteria, representing non-pathogens were reduced in both non-severe and severe asthmatic groups compared to the healthy group, while Firmicutes were markedly increased in severe asthmatics. Amongst the Firmicutes, we also found that 2 *Streptococcus* spp., *Streptococcus*_23 and *Streptococcus*_155, were correlated with asthma severity. In addition, one OTU identified as *Prevotella* spp. showed negative correlation with asthma status. Though alpha diversity measures did not demonstrate a difference between asthma subgroups, there were small significant differences in the whole community structure as determined using an analysis of beta diversity. Thus, the airway microbiota in severe asthma is different from the non-severe asthma patient, which is in turn different from healthy controls. Furthermore, non-severe asthmatics demonstrated an increase in Proteobacteria relative to either healthy controls or severe asthmatics.

In the study of Marri et al [38], who also used induced sputum samples from 10 non-asthmatic and 10 adults with asthma not taking inhaled or oral corticosteroid therapy at the time of the study, a greater abundance of Proteobacteria, particularly Haemophilus, and lower abundance of Firmicutes was reported. The increase in Proteobacteria confirmed the results of earlier studies [12, 13] using bronchial brushings from asthma patients who were on inhaled corticosteroid treatment, where Haemophilus was particularly seen to be increased, with the former study also reporting a decrease in Prevotella. Furthermore, our previous study [12], a group of asthmatic children with difficult-to-treat asthma on high dose inhaled corticosteroids, an increase in Proteobacteria/Haemophilus and Firmicutes/Staphylococcus or Streptococcus, and a decrease on Bacteroidetes/Prevotella were observed in bronchoalveolar lavage fluid samples. Our current data focused on an analysis of sputum samples from patients with severe asthma are consistent with the differences in phyla/genera previously reported between asthma and controls. Recently, using bronchial brushings from the lower airways, Huang et al. have reported that patients with severe asthma were significantly enriched in Actinobacteria compared to healthy control subjects or patients with mild-to-moderate asthma, with the largest differences seen in a member of the Klebsiella genus [39]. Whether the discrepancy between our findings and that of Huang et al. is due to differences in the region of the lung is unclear at present and would necessitate further investigation whereby intra-subject comparisons are done of bronchial brushing versus induced sputum.

Asthma is a heterogeneous syndrome resulting from an interaction of different environmental factors [such as allergens, smoking and medications] with host factors to produce complex phenotypic manifestations [3, 27]. Phenotypic complexity can be simplified by statistical procedures such as principal component analysis [PCA] which identifies the most important variables and their correlations. PCA of the phenotypic data led to the definition of four main principal components. The first 2 clusters had characteristics of severe asthma [3] and were associated with the 3 bacterial species that were over- or under-represented in severe asthma. Thus, in Cluster PC2, patients on oral corticosteroid therapy with an atopic background, sputum eosinophilia and airflow obstruction were more likely to be associated with an increase of *Streptococcus*_155 and a decrease in *Prevotella*_292. On the other hand, Cluster PC1 also representing a severe group not on oral corticosteroids but on high dose inhaled corticosteroids and long-acting β-agonist, with a long duration of asthma and non-atopic background, was associated with an increase in both *Streptococcus* _23 and *Streptococcus*_155, and to some extent with a reduction in *Prevotella*_292. The greatest association with an increase in *Streptococcus*_155 and *Streptococcus*_23 was seen in Cluster PC3, characterised by both blood and sputum eosinophilia, but with a lower sputum neutrophil count, and nasal disease, with an increased likelihood of being on oral corticosteroids. These associations in distinct clusters of asthma may reflect the influence of specific elements of the microbiota on well-known characteristics of severe asthma such as eosinophilia, chronic airflow obstruction and corticosteroid insensitivity [40]. Alternatively, asthma treatments [such as corticosteroid or β-adrenergic agonist therapies] or the differences between asthma subtypes may select for particular microorganisms.

Several confounding factors particularly in the severe asthmatic subjects need to be taken into consideration. First, the effect of asthma treatments on the airway microbiota remains unclear. In addition to potential unknown effects on bacterial persistence and growth, these medications may alter the innate immune response to bacteria that may in turn determine their pathogenicity. By definition, the patients with severe asthma that we studied were already on high doses of inhaled corticosteroids, often on oral corticosteroids. However, none were on antibiotic therapy or had been in the past 6 weeks, as this was one of the major entry criteria. The potential effect of corticosteroids on the lung microbiota is unclear, but comparing the microbiota data of Marri et al. [38] where patients with asthma not on inhaled corticosteroids were selected with those of Hilty et al. [12], there were no major differences that would indicate an effect of inhaled corticosteroids. On the other hand, in the small study of the lung microbiome in patients with moderate to severe COPD, the alterations in lung microbiome reported may have been accounted for by inhaled corticosteroid therapy [41]. The other important observation is that the gut microbiota has been reported to completely metabolise corticosteroids such as prednisolone, beclomethasone dipropionate and budesonide, but the effect of the airway microbiota in this context remains unknown [42]. The possibility that such an action by selected bacteria to preferentially metabolise corticosteroids could underlie corticosteroid insensitivity in severe asthma has not been tested.

The significance of the changes in sputum microbiota that we observed in severe asthma suggests the need for further investigation. Since our patients with severe asthma may be classified as being corticosteroid-insensitive as demonstrated by studies on peripheral blood mononuclear cells or alveolar macrophages [43, 44], the recent study by Goleva and colleagues on bronchoalveolar lavage microbiota of patients defined as corticosteroid-resistant and corticosteroid insensitive is of interest [23]. In corticosteroid-resistant patients, they reported an increase in the proportion of sequences of microorganisms in the phyla Actinobacteria and Proteobacteria, and reduced sequences for the genera *Prevotella* and *Veillonella*, and Phylum Fusobacteria compared to normal control subjects [23]. Our data are partly in agreement with the reported reduction in Prevotella. Goleva et al further showed that the bacterium that was overexpressed in these patients, *Haemophilus parainfluenza*e, can directly induce corticosteroid resistance. In addition, Th-17 cells may be induced by bacterial infections and this has been implicated in corticosteroid insensitivity [45, 46].

Interestingly, the sputum culture results did not correlate with what was observed from the 16s rRNA analysis with positive cultures of *Haemophilus influenzae*, *Moraxella catarrhalis* and *Staphylococcus aureus* being most commonly observed in the asthmatic samples. This may not be surprising given the fact that sputum culture results will depend to a large extent on the media on which the bacteria are grown. On the other hand, DNA-based assays of microbiota does not allow one to determine the activity of members of the microbial community. A more comprehensive approach to culturing these bacteria is needed.

One potential weakness of our study is the inevitable contamination of the upper airway microflora in the sputum samples. However, this contamination would have been similar for all subjects although the upper airway microbiota would also be influenced by the oral and dental state and the presence of comorbidities such as rhinosinusitis, laryngitis or gastrooesophageal reflux. The microbiota we found in this study is quite distinct from that described in various compartments of the upper airway and oral cavity such as saliva, nasopharynx and nose [47, 48]. Even in studies that have directly sampled the lower airways through a bronchoscope for brushings, biopsies or bronchoalveolar lavage fluid, there may have been contamination brought on by the passage of the bronchoscope through the upper airways prior to getting to the lower airways. At the other extreme, there is the suggestion that the bacteria found in the lower airway represents contamination of bacteria from the upper airways [49]. However, part of this microbiota is unique representing an adaptation towards proliferation in the lung environment [50]. It is important that such a study was performed in sputum samples because this is the most non-invasive way of obtaining lower airway samples relatively safely from patients, and will represent the only acceptable method of obtaining lower airway samples from all patients with asthma.

While the 16S rRNA gene sequencing approach to define microbiota provides us with the bacterial members of the airway microbiota, it does not provide us with the fungi and viruses potentially present in the lower airways. Their potential contribution therefore is missed by using this approach. Another limitation of our cross-sectional study is that we are looking at a single time point and therefore are restricted in terms of the dynamic nature of the microbiota in severe asthma. Longitudinal studies would be advantageous but far from trivial when balancing the clinical care of patients with such severe disease and repeated invasive sampling. In addition, dormancy is common in microbial communities and activity of community members ideally requires a direct measure of activity such as RNA, protein or metabolic assays or serial sampling (requiring longitudinal studies) that we were unable to perform here. Finally, there are other alternative analytical methods available in the microbiome literature that could have been used in our study, but we have here restricted ourselves to the most commonly used methods in the absence of bench-marking studies. For example, there are many different and novel statistical tests available for comparing OTUs and other phylogenetic levels in microbiome studies, but no bench-marking. We have therefore resorted to use a well-established statistical test corrected for multiple testing.

In summary, we have shown that in severe asthma, there is a distinct alteration of the sputum microbiota with a greater prominence of Firmicutes (including 2 Streptococcal OTUs, and a lower prominence of Bacteroidetes, particularly Prevotella spp. These bacterial OTUs were related to distinct components of the severe asthma syndrome, particularly those relating to the use of oral corticosteroids. Analysis of 16S rRNA gene sequences differentiates poorly between different streptococcal species, and further classification by typing additional polymorphic loci [18] as well as cultures of streptococcal isolates is desirable in future studies. In addition, the potential role of these streptococcal species in the pathogenesis of severe asthma will need further investigation.
